# Robust Distributed Observers for Simultaneous State and Fault Estimation over Sensor Networks

**DOI:** 10.3390/s24237589

**Published:** 2024-11-27

**Authors:** Dingguo Liang, Yunxiao Ren, Yuezu Lv, Silong Wang

**Affiliations:** 1 Department of Mechanics and Engineering Science, College of Engineering, Peking University, Beijing 100871, China; liangdg0915@gmail.com (D.L.); renyx@pku.edu.cn (Y.R.); 2 Advanced Research Institute of Multidisciplinary Sciences, Beijing Institute of Technology, Beijing 100081, China; yzlv@bit.edu.cn; 3 Deep Space Exploration Laboratory, Beijing 100195, China

**Keywords:** distributed observers, sensor networks, fault estimation, *H*_∞_ performance, spacecraft attitude control

## Abstract

This paper focuses on simultaneous estimation of states and faults for a linear time-invariant (LTI) system observed by sensor networks. Each sensor node is equipped with an observer, which uses only local measurements and local interaction with neighbors for monitoring. The observability of said observer is analyzed where non-local observability of a sensor node is required in terms of the system state and faults. The distributed observers present features of $H_{\infty }$ performance to constrain the influence of disturbances on the estimation errors, for which the global design condition is transformed into a linear matrix inequality (LMI). The LMI is proven to be solvable given collective observability of the system and a suitable $H_{\infty }$ performance index. Moreover, in the case that no disturbances exist, fully distributed observers with adaptive gains are designed to asymptotically estimate the states and faults without using any global information from the network. Finally, the effectiveness of the proposed methods is verified through case studies on a spacecraft’s attitude control system.

## 1. Introduction

Nowadays, distributed state estimation is paid plenty of attention for monitoring the state of a target system over spatially deployed multi-agents or sensor networks [1,2,3,4,5,6,7,8,9,10,11,12,13,14] and has been applied in many settings, such as cooperative tracking and monitoring [15,16,17,18], environmental exploration [19,20] and cyber security [21,22]. In these scenarios, sensors play an important role for monitoring the state of the target system, and the estimated state can help perform higher-level tasks such as feedback stabilization and decision making. The core feature of distributed state estimation is that each sensor only has access to a portion of the complete output such that the state can not be successfully estimated by a single sensor. By cooperatively exchanging estimation information over a wireless communication network, however, all of the sensors can completely estimate the state. Thus, no global information is needed for the sensor network, and the state estimation can be implemented in a distributed way.

Distributed state estimation is usually subdivided into two directions: a distributed filtering-based approach and a distributed observer-based approach. In the distributed filtering framework [1,2,3,4], the objective is to estimate the state of the target system in the presence of Gaussian noise such that the covariance is minimized. On the other hand, distributed observers [5,6,7,8,9,10,11,12,13,14] aim mainly for the stabilization of the estimation error, which is more suitable for dealing with certain deterministic disturbances through the observer gain design. Ref. [5] proposed a solvable LMI-based design of distributed observers for a continuous-time LTI system such that the estimation error could converge to zero at any a priori given decay rate. Based on detectability decomposition, ref. [6] proposed a decentralized design for the distributed observers, where each sensor could recover its own detectable part while the undetectable part was compensated for using estimation information exchanged over sensor networks. To handle disturbances in the process, ref. [7] designed fully distributed, reduced-order, unknown input observers using adaptive gains. To explore the robustness of the distributed observers, ref. [8] considered $H_{2}$ and $H_{\infty }$ suboptimal distributed estimation problems, which were solved using a centralized LMI-based method. In [11], the authors extended their work in [23] on the design of asymptotic-convergence unknown input observers via an interval observer to the case of distributed estimation, where asymptotic estimations of the system state, unknown input and measurement noise could be obtained simultaneously. Ref. [12] investigated the estimation problem for a class of Lipschitz nonlinear systems, where redundant sensors were implemented such that through the selection of fault-free sensor signals, the designed distributed fault-tolerant observer could estimate the system states and actuator faults.

In practice, with the growth in the complexity of control systems, faults are more likely to occur and thus damage the performance of the system [24,25]. For distributed observers over sensor networks, process faults and even sensor faults in a single node can both damage the whole network since faulty information can be broadcasted to other nodes by the communication topology [26]. Hence, the fault diagnosis of distributed observers should be paid more attention. Though faults may be handled in a decoupled or robust manner when handling disturbances as shown in [7,8,11], the assumptions for disturbances in [7,8,11] are usually not satisfied for general faults. To realize fault-tolerant control of the target system, accurate fault information still should be known, and thus fault estimation within distributed observers is required [27]. Though [11] proposed unknown input reconstruction which can be applied to fault estimation, this method is only applied to actuator faults and requires a strict rank condition. So, the existing works on distributed observers [5,6,7,8,9,10,11,12,13,14] cannot be directly applied to the general case of distributed fault estimation, and some other conditions and methods should be introduced.

Based on the aforementioned discussion, this paper investigates the problem of simultaneous distributed estimation of states and faults for a continuous-time LTI system observed by sensor networks. The main contributions are given as follows:

1. A set of robust distributed observers is proposed for the target system, where $H_{\infty }$ performance is introduced such that the state and faults can be asymptotically estimated with a capacity for disturbance attenuation. The design of the distributed observers requires not local observability of the system state or faults in a single node but rather collective observability of the state and faults.
2. Compared with [8], the proposed $H_{\infty }$ distributed observers are optimal and solved using an LMI; thus, a minimized $H_{\infty }$ performance index can be computed. Meanwhile, rigorous derivation proves that such an LMI must have a solution given the collective observability condition.
3. In the case that no disturbances exist, a fully distributed method is designed for estimating the state and faults, where each observer is equipped with the adaptive gain relevant only to local and neighboring estimation such that no global information is required for either the distributed observer construction or online running.

The rest of this paper is organized as follows: Section 2 presents the preliminaries on the graph theory and problem formulation. Section 3 considers the design of the distributed observers for state and fault estimation, where robust distributed observers with $H_{\infty }$ performance and fully distributed observers with adaptive gains are proposed successively. In Section 4, the effectiveness of the proposed methods is illustrated through case studies on a spacecraft attitude control system. Finally, Section 5 concludes this paper.

**Notations:**

0 and *I* denotes the zero and identity matrices with approximate dimensions. Define M={1,2,…,M}; then, for a set of vectors or matrices $\left\{A_{i}\in R^{m_{i}\times n}|i\in M\right\}$, the concatenated form is defined as $\mathrm{col}{\left(A_{i}\right)}_{i\in M}={\left[A_{1}^{T},A_{2}^{T},\ldots ,A_{M}^{T}\right]}^{T}$; for a set of matrices $\left\{B_{i}\in R^{m_{i}\times n_{i}}|i\in M\right\}$, the block diagonal form is defined as $\mathrm{diag}{\left(B_{i}\right)}_{i\in M}$. For a square matrix *A*, $\lambda_{max}\left(A\right)$$\left(\lambda_{min}\left(A\right)\right)$ denotes the largest (smallest) real part of the eigenvalues of *A* and $\mathrm{Sym}\left(A\right)=A+A^{T}$. The Kronecker product of the matrices $A_{1}$ and $A_{2}$ is denoted by $A_{1}⊗A_{2}$. ${∥\cdot ∥}_{2}=\sqrt{\int_{0}^{\infty }{∥\cdot ∥}^{2}dt}$ denotes the $L_{2}$-norm and ∥·∥ denotes the Euclidean norm.

ImA and KerA are, respectively, the image and the kernel of *A*. $\chi_{A}\left(s\right)=\chi_{A}^{+}\left(s\right)\chi_{A}^{-}\left(s\right)$ is the characteristic polynomial of *A*, where the roots of $\chi_{A}^{+}$ and $\chi_{A}^{-}$ belong to the open left and the closed right half-planes of the complex plane, respectively. $U\left(C,A\right)=\left({⋂}_{k=1}^{n}\mathrm{Ker}CA^{k-1}\right)$ denotes the unobservable subspace of the pair (C,A).

## 2. Preliminaries and Problem Formulation

Communication among the sensor network is described by a directed strongly connected graph $G=\left\{V,E\right\}$ of *N* nodes, where V={1,2,…,N} is the vertex set, with i∈V corresponding to node *i*, and E⊆V×V is the edge set of the graph. The neighborhood set of node $V_{i}$ is denoted by $N_{i}=\left\{V_{j}\in V:\left(V_{i},V_{j}\right)\in E,i\neq j\right\}$. The adjacent matrix of graph G is denoted by $A=\left[a_{ij}\right]\in R^{N\times N}$, where $a_{ij}>0$ if $(V_{i},V_{j})\in E$ and $a_{ij}=0$ otherwise. A directed graph is said to be strongly connected if there exists a path from every node to every other node. The Laplacian matrix of graph G is defined as L = $\left[l_{ij}\right]\in R^{N\times N}$, whose entries are $l_{ii}$ = $\sum_{j=1}^{N}a_{ij}$ and $l_{ij}$ = $-a_{ij}$, *i* ≠ *j*.

Let a network of *N* sensors together monitor the continuous-time LTI system, with the dynamics described as

(1) $$\begin{array}{cc} \; & \dot{x}=Ax+Bu+E_{0}f+D_{0}d_{0}\\ \; & y_{i}=C_{i}x+E_{i}f+D_{i}d_{i}\;i\in V \end{array}$$

where $x\in R^{n}$ is the state vector of the system, $u\in R^{l}$ is the control input, $y_{i}\in R^{p_{i}}$ is the output in the *i*th sensor node, $f\in R^{m}$ is the possible fault influencing both state *x* and output $y_{i}$ and $d_{0}\in R^{q_{0}}$ and $d_{i}\in R^{q_{i}}$ are the process disturbance and measurement disturbance in the *i*th sensor node, respectively. Meanwhile, $A_{i}\in R^{n\times n},B_{i}\in R^{n\times l},E\in R^{n\times m},D_{0}\in R^{n\times q_{0}},C_{i}\in R^{p_{i}\times n},E_{i}\in R^{p_{i}\times m},D_{i}\in R^{p_{i}\times q_{i}}$ are known constant matrices.

For simultaneous detection of the state *x* and fault *f*, consider an augmented state vector as $\bar{x}={\left[\begin{array}{cc} x^{T} & f^{T} \end{array}\right]}^{T}\in R^{\bar{n}},$ where $\bar{n}=n+m$. Then, the dynamics equivalent to (1) with respect to $\bar{x}$ is formulated as follows:

(2) $$\begin{array}{cc} \; & \dot{\bar{x}}=\bar{A}\bar{x}+\bar{B}u+Ww\\ \; & y_{i}={\bar{C}}_{i}\bar{x}+D_{i}d_{i},\;i\in V \end{array}$$

where $w={\left[\begin{array}{cc} d_{0}^{T} & {\dot{f}}^{T} \end{array}\right]}^{T}\in R^{{\bar{q}}_{0}}$ with ${\bar{q}}_{0}=q_{0}+m$, $\bar{A}=\left[\begin{array}{cc} A & E_{0}\\ 0 & 0 \end{array}\right]$, $\bar{B}=\left[\begin{array}{c} B\\ 0 \end{array}\right]$, $W=\left[\begin{array}{cc} D_{0} & 0\\ 0 & I \end{array}\right]$, ${\bar{C}}_{i}=\left[\begin{array}{cc} C_{i} & E_{i} \end{array}\right]$.

The following assumptions are needed throughout this paper.

**Assumption 1.**  *System (2) possesses collective observability, that is, the pair $\left(\mathrm{col}{\left({\bar{C}}_{i}\right)}_{i\in V},\bar{A}\right)$ is observable.*

**Assumption 2.**  *The fault f is differentiable at any time with its derivative denoted as $\dot{f}$. Meanwhile, $\dot{f}$, $d_{0}$ and $d_{i}$ are $L_{2}$-norm bounded for i∈V.*

Define $\bar{C}=\mathrm{col}{\left({\bar{C}}_{i}\right)}_{i\in V}$; then, the observability matrix of the pair $\left(\mathrm{col}{\left({\bar{C}}_{i}\right)}_{i\in V},\bar{A}\right)$ is given as

$$O=\left[\begin{array}{c} \bar{C}\\ \bar{C}\bar{A}\\ \vdots \\ \bar{C}{\bar{A}}^{\bar{n}-1} \end{array}\right].$$

Lemma 1 is then presented to achieve a deeper understanding of Assumption 1.

**Lemma 1.**  *The pair $\left(\mathrm{col}{\left({\bar{C}}_{i}\right)}_{i\in V},\bar{A}\right)$ is observable if and only if the following two conditions are satisfied:*

*(1) The pair $\left(\mathrm{col}{\left(C_{i}\right)}_{i\in V},A\right)$ is observable;*

*(2) $O_{2}$ is of full column rank, and*

(3) $$\begin{array}{c} \mathrm{rank}\left[\begin{array}{cc} O_{1} & O_{2} \end{array}\right]=\mathrm{rank}\left(O_{1}\right)+\mathrm{rank}\left(O_{2}\right) \end{array}$$

*where $O_{1}=\left[\begin{array}{c} C\\ CA\\ \vdots \\ CA^{n-1} \end{array}\right]$ and $O_{2}=\left[\begin{array}{c} E_{0}\\ C\;E\\ \vdots \\ CE^{n-1} \end{array}\right]$, with $C=\mathrm{col}{\left(C_{i}\right)}_{i\in V}$ and $E=\mathrm{col}{\left(E_{i}\right)}_{i\in V}$.*

**Proof.**  It is obvious that the observability of $\left(\mathrm{col}{\left({\bar{C}}_{i}\right)}_{i\in V},\bar{A}\right)$ is equivalent to O being of full column rank. In view of (2),

$$O=\left[\begin{array}{c} \bar{C}\\ \bar{C}\bar{A}\\ \vdots \\ \bar{C}{\bar{A}}^{\bar{n}-1} \end{array}\right]=\left[\begin{array}{cc} C & E_{0}\\ CA & C\;E\\ \vdots & \vdots \\ CA^{\bar{n}-1} & CE^{\bar{n}-1} \end{array}\right]=\left[\begin{array}{cc} {\bar{O}}_{1} & {\bar{O}}_{2} \end{array}\right]$$

where ${\bar{O}}_{1}=\left[\begin{array}{c} C\\ CA\\ \vdots \\ CA^{\bar{n}-1} \end{array}\right]$ and ${\bar{O}}_{2}=\left[\begin{array}{c} E_{0}\\ C\;E\\ \vdots \\ CE^{\bar{n}-1} \end{array}\right]$.

It can be easily deduced that $\mathrm{rank}\left(O_{1}\right)=\mathrm{rank}\left({\bar{O}}_{1}\right)$ and $\mathrm{rank}\left(O_{2}\right)=\mathrm{rank}\left({\bar{O}}_{2}\right)$, according to the property of the observability matrix. Then, one has

$$\mathrm{rank}\left(O\right)=\mathrm{rank}\left[\begin{array}{cc} {\bar{O}}_{1} & {\bar{O}}_{2} \end{array}\right]=\mathrm{rank}\left[\begin{array}{cc} O_{1} & O_{2} \end{array}\right].$$

Thus, $\mathrm{rank}\left(O\right)=\bar{n}$ if and only if $\mathrm{rank}(O_{1})=n$, $\mathrm{rank}(O_{2})=m$, as well as (3) being satisfied, which is equivalent to conditions (1) and (2). This ends the proof. □

Since for each node $\left({\bar{C}}_{i},\bar{A}\right)$ is not necessarily observable, observability decomposition for $\left({\bar{C}}_{i},\bar{A}\right)$ is needed to facilitate the design of distributed observers. Let $\rho_{i}$ be the dimension of $U({\bar{C}}_{i},\bar{A})$ and $U{\left({\bar{C}}_{i},\bar{A}\right)}^{⊥}$ be the orthogonal complement of $U({\bar{C}}_{i},\bar{A})$; then, we introduce an orthonormal transformation matrix $T_{i}\in R^{\bar{n}\times \bar{n}}$ as $T_{i}=\left[\begin{array}{cc} O_{i} & U_{i} \end{array}\right]$, where $U_{i}\in R^{\bar{n}\times \rho_{i}}$ and $O_{i}\in R^{\bar{n}\times \left(\bar{n}-\rho_{i}\right)}$ are matrices whose columns are orthonormal bases of $U({\bar{C}}_{i},\bar{A})$ and $U{\left({\bar{C}}_{i},\bar{A}\right)}^{⊥}$, respectively. Meanwhile, set $T=\mathrm{diag}{\left(T_{i}\right)}_{i\in V}$, $O=\mathrm{diag}{\left(O_{i}\right)}_{i\in V}$ and $U=\mathrm{diag}{\left(U_{i}\right)}_{i\in V}$. The observability decomposition corresponding to the pair $\left({\bar{C}}_{i},\bar{A}\right)$ is derived as

(4) $$T_{i}^{T}\bar{A}T_{i}=\left[\begin{array}{cc} {\bar{A}}_{io} & 0\\ {\bar{A}}_{ir} & {\bar{A}}_{iu} \end{array}\right],\;{\bar{C}}_{i}T_{i}=\left[\begin{array}{cc} {\bar{C}}_{io} & 0 \end{array}\right],$$

where the pair $\left(\mathrm{col}{\left({\bar{C}}_{io}\right)}_{i\in V},{\bar{A}}_{io}\right)$ is observable. Accordingly, *W* is partitioned as

(5) $$T_{i}^{T}W=\left[\begin{array}{c} W_{io}\\ W_{iu} \end{array}\right]$$

## 3. Main Results

### 3.1. Distributed Observer Design for State and Fault Estimation

In this subsection, the distributed observers are designed for dynamics (2). Note that in a distributed case, the observer in each node needs to make full use of local measurement and estimation from its neighbors. Then, sensor node *i* is equipped with the observer in the following form:

(6) $${\dot{\hat{\bar{x}}}}_{i}=\bar{A}{\hat{\bar{x}}}_{i}+K_{i}\left(y_{i}-{\bar{C}}_{i}{\hat{\bar{x}}}_{i}\right)+H_{i}\sum_{j=1}^{N}a_{ij}\left({\hat{\bar{x}}}_{j}-{\hat{\bar{x}}}_{i}\right),\;i\in V$$

where ${\hat{\bar{x}}}_{i}\in R^{\bar{n}}$ is the estimated state of $\bar{x}$ in the *i*-th sensor node, and matrices $K_{i}$ and $H_{i}$ are gain matrices to be designed.

**Figure 1:**
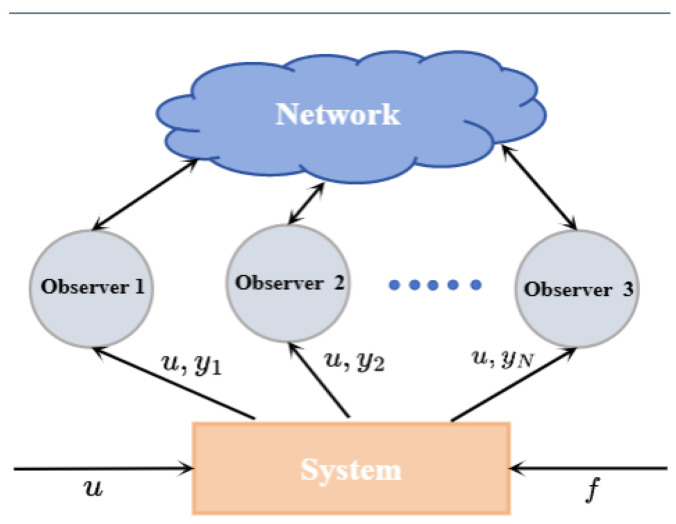
Schematic diagram of distributed observer design.

A schematic diagram of the distributed observer design is shown in Figure 1.

Then, the local estimation error of the observer in sensor node *i* is defined as ${\bar{e}}_{i}={\hat{\bar{x}}}_{i}-x$, which, in substituting (6) into (2), has the following dynamics:

(7) $${\dot{\bar{e}}}_{i}=\left(\bar{A}-K_{i}{\bar{C}}_{i}\right){\bar{e}}_{i}+H_{i}\sum_{j=1}^{N}a_{ij}\left({\bar{e}}_{j}-{\bar{e}}_{i}\right)+\left({\bar{W}}_{i}-K_{i}{\bar{D}}_{i}\right){\bar{w}}_{i}\;$$

where ${\bar{w}}_{i}={\left[\begin{array}{cc} w^{T} & d_{i}^{T} \end{array}\right]}^{T}\in R^{{\bar{q}}_{i}}$ with ${\bar{q}}_{i}={\bar{q}}_{0}+q_{i}$, ${\bar{W}}_{i}=\left[\begin{array}{cc} W & 0 \end{array}\right]$ and ${\bar{D}}_{i}=\left[\begin{array}{cc} 0 & D_{i} \end{array}\right]$.

Define $\bar{e}=\mathrm{col}{\left({\bar{e}}_{i}\right)}_{i\in V}$ as the concatenated estimation error and $\bar{w}=\mathrm{col}{\left({\bar{w}}_{i}\right)}_{i\in V}$ as the concatenated disturbances. Thus, one has the following global estimation error dynamics:

(8) $$\dot{\bar{e}}=\left(A-KC\right)\bar{e}-H\left(L⊗I_{\bar{n}}\right)\bar{e}+\left(W-KD\right)\bar{w}$$

where $A=I_{N}⊗\bar{A}$, $K=\mathrm{diag}{\left(K_{i}\right)}_{i\in V}$, $C=\mathrm{diag}{\left({\bar{C}}_{i}\right)}_{i\in V}$, $H=\mathrm{diag}{\left(H_{i}\right)}_{i\in V}$, $W=\mathrm{diag}{\left({\bar{W}}_{i}\right)}_{i\in V}$ and $D=\mathrm{diag}{\left({\bar{D}}_{i}\right)}_{i\in V}$.

Before presenting the following results, Lemma 2 is needed.

**Lemma 2.**  *Suppose that $G=\left\{V,E\right\}$ is strongly connected and L is the associated Laplacian matrix; then, positive scalars $\theta_{i}$ exist for i∈V satisfying $\sum_{1}^{N}\theta_{i}=1$ and $\mathrm{col}{\left(\theta_{i}\right)}_{i\in V}^{T}L=0$. Moreover, we define $\hat{L}=\theta L+L^{T}\theta$ with $\theta =\mathrm{diag}{\left(\theta_{i}\right)}_{i\in V}$. Under Assumption 1, the following two statements are satisfied:*

*(1) For all $r_{i}>0$, i∈V, there exists ε>0 satisfying*

(9) $$T^{T}\left(\hat{L}⊗I_{n}\right)T+R>\varepsilon I\;$$

*where $R=\mathrm{diag}{\left(R_{i}\right)}_{i\in V}$ with*

$$R_{i}=\left[\begin{array}{cc} r_{i}I_{\rho_{i}} & 0\\ 0 & 0_{n-\rho_{i}} \end{array}\right],i\in V.$$

*(2) $U^{T}\left(L⊗I_{n}\right)U$ is nonsingular, and $U^{T}\left(\hat{L}⊗I_{n}\right)U>0$.*

**Proof.**  This lemma is a combination of Lemma 4 in [5] and Lemma 4 in [6], which is thus directly obtained. □

### 3.2. Robust Distributed Observers with $H_{\infty }$ Performance

In this subsection, the objective of the distributed observer in each node is to cooperatively estimate the augmented state $\bar{x}$ in the presence of disturbances ${\bar{w}}_{i}$. Thus, distributed observers (6) with $H_{\infty }$ performance are needed to attenuate the influence of disturbances $\bar{w}$ on the estimation error $\bar{e}$, which is defined as follows:

**Definition 1.**  *For a given positive scalar γ, distributed observers (6) for system (2) are said to be robust with the $H_{\infty }$ performance index γ, if the global estimation error dynamics (8) is asymptotically stable and satisfies the following $H_{\infty }$ disturbance attenuation condition:*

(10) $$\begin{array}{c} \int_{0}^{\infty }{\bar{e}}^{T}\bar{e}dt\leq \gamma^{2}\int_{0}^{\infty }{\bar{w}}^{T}\bar{w}dt \end{array}$$

Hence, to maximize the $H_{\infty }$ performance, the main objective in this subsection is to find the smallest γ satisfying Definition 1. Theorem 1 is therefore presented to design appropriate gain matrices $K_{i}$, $H_{i}$ for i∈V and scalar γ such that distributed observers (6) are robust with $H_{\infty }$ performance.

**Theorem 1.**  *Under Assumptions 2, the error dynamics (8) is asymptotically stable and satisfies the $H_{\infty }$ performance (10), if there exist positive scalars γ, κ, matrices $P_{io}>0$, $P_{iu}>0$, ${\tilde{K}}_{io}$ such that the following LMI holds*

(11) $$\Psi =\left[\begin{array}{cccc} \Psi_{11} & \Psi_{12} & \cdots & \Psi_{1N}\\ {}* & \Psi_{22} & \cdots & \Psi_{2N}\\ {}* & * & \ddots & \vdots \\ {}* & * & * & \Psi_{NN} \end{array}\right]<0,$$

*where*

(12) $$\Psi_{ii}=\left[\begin{array}{cccc} \mathrm{Sym}\left(P_{io}{\bar{A}}_{io}-{\tilde{K}}_{io}{\bar{C}}_{io}\right)+\left(1-2\kappa \theta_{i}l_{ii}\right)I & {\left(P_{iu}{\bar{A}}_{ir}\right)}^{T} & P_{io}W_{io} & -{\tilde{K}}_{io}D_{i}\\ {}* & \mathrm{Sym}\left(P_{iu}{\bar{A}}_{iu}\right)+\left(1-2\kappa \theta_{i}l_{ii}\right)I & P_{iu}W_{iu} & 0\\ {}* & * & -\gamma^{2}I & 0\\ {}* & * & * & -\gamma^{2}I \end{array}\right]$$

(13) $$\Psi_{ij}=\left[\begin{array}{cc} -\kappa \left(\theta_{i}l_{ij}+\theta_{j}l_{ji}\right)T_{i}^{T}T_{j} & 0\\ 0 & 0 \end{array}\right]$$

*for i,j=1,…,N,i≠j.*

*Then, the optimal $H_{\infty }$ performance index γ is obtained by solving the following optimization problem.*

(14) $$\begin{array}{c} \begin{array}{cc} \min & \gamma \\ \;s.t.\; & \left(11\right) \end{array} \end{array}$$

*Moreover, gain matrices $K_{i}$ and $H_{i}$ for i∈V are given as*

(15) $$\begin{array}{c} K_{i}=T_{i}\left[\begin{array}{c} P_{io}^{-1}{\tilde{K}}_{io}\\ 0 \end{array}\right],\;H_{i}=\kappa \theta_{i}T_{i}\left[\begin{array}{cc} P_{io}^{-1} & 0\\ 0 & P_{iu}^{-1} \end{array}\right]T_{i}^{T}. \end{array}$$

**Proof.**  A Lyapunov function candidate is given as

(16) $$\begin{array}{c} V=\sum_{i=1}^{N}{\bar{e}}_{i}^{T}P_{i}{\bar{e}}_{i}\; \end{array}$$

where $P_{i}=T_{i}\left[\begin{array}{cc} P_{io} & 0\\ 0 & P_{iu} \end{array}\right]T_{i}^{T}$.

According to (7) and by using (4), (5) and (15), the time derivative of *V* has the following form:

(17) $$\begin{array}{c} \begin{array}{cc} \; & \dot{V}=\sum_{i=1}^{N}\left({\dot{\bar{e}}}_{i}^{T}P_{i}{\bar{e}}_{i}+{\bar{e}}_{i}^{T}P_{i}{\dot{\bar{e}}}_{i}\right)\\ & =2\sum_{i=1}^{N}{\bar{e}}_{i}^{T}P_{i}\left(\left(\bar{A}-K_{i}{\bar{C}}_{i}\right){\bar{e}}_{i}+H_{i}\sum_{j=1}^{N}a_{ij}\left({\bar{e}}_{j}-{\bar{e}}_{i}\right)+\left({\bar{W}}_{i}-K_{i}{\bar{D}}_{i}\right){\bar{w}}_{i}\right)\\ & =2\sum_{i=1}^{N}{\bar{e}}_{i}^{T}T_{i}\left(\left[\begin{array}{cc} P_{io}{\bar{A}}_{io}-{\tilde{K}}_{io}{\bar{C}}_{io} & 0\\ P_{iu}{\bar{A}}_{ir} & P_{iu}{\bar{A}}_{iu} \end{array}\right]T_{i}^{T}{\bar{e}}_{i}\right)-2\sum_{i=1}^{N}{\bar{e}}_{i}^{T}\left(\kappa \theta_{i}\sum_{j=1}^{N}l_{ij}{\bar{e}}_{j}\right)\\ & +2\sum_{i=1}^{N}{\bar{e}}_{i}^{T}T_{i}\left[\begin{array}{cc} P_{io}W_{io} & -{\tilde{K}}_{io}D_{i}\\ P_{iu}W_{iu} & 0 \end{array}\right]{\bar{w}}_{i} \end{array} \end{array}$$

It is not difficult to find from (11) that

(18) $$\Phi =\left[\begin{array}{cccc} \Phi_{11} & \Phi_{12} & \cdots & \Phi_{1N}\\ {}* & \Phi_{22} & \cdots & \Phi_{2N}\\ {}* & * & \ddots & \vdots \\ {}* & * & * & \Phi_{NN} \end{array}\right]<0,$$

where $\Phi_{ii}=\left[\begin{array}{cc} \mathrm{Sym}\left(P_{io}{\bar{A}}_{io}-{\tilde{K}}_{io}{\bar{C}}_{io}\right)+\left(1-2\kappa \theta_{i}l_{ii}\right)I & {\left(P_{iu}{\bar{A}}_{ir}\right)}^{T}\\ {}* & \mathrm{Sym}\left(P_{iu}{\bar{A}}_{iu}\right)+\left(1-2\kappa \theta_{i}l_{ii}\right)I \end{array}\right]$, $\Phi_{ij}=-\kappa \left(\theta_{i}l_{ij}+\theta_{j}l_{ji}\right)T_{i}^{T}T_{j}$ for i,j=1,…,N,i≠j.

Note that I>0. Thus, in the absence of disturbances ${\bar{w}}_{i}$ for i∈V, pre- and post- multiplying (18) with ${\bar{e}}^{T}T$ and its transpose, respectively, we yield

$$\dot{V}=2\sum_{i=1}^{N}{\bar{e}}_{i}^{T}T_{i}\left(\left[\begin{array}{cc} P_{io}{\bar{A}}_{io}-{\tilde{K}}_{io}{\bar{C}}_{io} & 0\\ P_{iu}{\bar{A}}_{ir} & P_{iu}{\bar{A}}_{iu} \end{array}\right]T_{i}^{T}{\bar{e}}_{i}\right)-2\sum_{i=1}^{N}{\bar{e}}_{i}^{T}\left(\kappa \theta_{i}\sum_{j=1}^{N}l_{ij}{\bar{e}}_{j}\right)<0$$

which means that the estimation error dynamics (8) is asymptotically stable.

Furthermore, we define ${\tilde{e}}_{i}=T_{i}{\bar{e}}_{i}$ and

(19) $$\begin{array}{c} J_{\infty }=\dot{V}+\sum_{i=1}^{N}\left({\bar{e}}_{i}^{T}{\bar{e}}_{i}-\gamma^{2}{\bar{w}}_{i}^{T}{\bar{w}}_{i}\right) \end{array}$$

In light of (17), $J_{\infty }$ is rewritten as

$$\begin{array}{cc} J_{\infty } & =2\sum_{i=1}^{N}{\tilde{e}}_{i}^{T}\left(\left[\begin{array}{cc} P_{io}{\bar{A}}_{io}-{\tilde{K}}_{io}{\bar{C}}_{io} & 0\\ P_{iu}{\bar{A}}_{ir} & P_{iu}{\bar{A}}_{iu} \end{array}\right]{\tilde{e}}_{i}\right)-2\sum_{i=1}^{N}{\tilde{e}}_{i}^{T}\left(\kappa \theta_{i}T_{i}^{T}\sum_{j=1}^{N}l_{ij}T_{j}{\tilde{e}}_{j}\right)\\ & +2\sum_{i=1}^{N}{\tilde{e}}_{i}^{T}\left[\begin{array}{cc} P_{io}W_{io} & -{\tilde{K}}_{io}D_{i}\\ P_{iu}W_{iu} & 0 \end{array}\right]{\bar{w}}_{i}+\sum_{i=1}^{N}\left({\tilde{e}}_{i}^{T}{\tilde{e}}_{i}-\gamma^{2}{\bar{w}}_{i}^{T}{\bar{w}}_{i}\right)\\ & =\eta^{T}\Psi \eta \end{array}$$

with $\eta =\mathrm{col}{\left({\tilde{e}}_{i}^{T},{\bar{w}}_{i}^{T}\right)}_{i\in V}^{T}$. From (11), one can obtain that $J_{\infty }<0$.

Then, under the zero initial condition, it is derived from (19) that

$$\begin{array}{c} \begin{array}{cc} \int_{0}^{\infty }J_{\infty }\;dt & =V\left(\infty \right)+\int_{0}^{\infty }\sum_{i=1}^{N}{\bar{e}}_{i}^{T}{\bar{e}}_{i}\;dt-\gamma^{2}\int_{0}^{\infty }{\bar{w}}_{i}^{T}{\bar{w}}_{i}\;dt \end{array} \end{array}$$

Note that V(∞)≥0; then, it is derived from $J_{\infty }<0$ that $\int_{0}^{\infty }{\bar{e}}^{T}\bar{e}dt\leq \gamma^{2}\int_{0}^{\infty }{\bar{w}}^{T}\bar{w}dt$, which indicates the $H_{\infty }$ performance. The proof is thus completed. □

To ensure the existence of distributed observers with $H_{\infty }$ performance, the solvability of the LMI (11) is analyzed in Theorem 2, which is relevant to the global observability assumed in Assumption 1.

**Theorem 2.**  *There always exist positive scalars γ, κ, matrices $P_{i1}>0$, $P_{i2}>0$, ${\tilde{K}}_{i1}$ such that LMI (11) holds if Assumption 1 holds.*

**Proof.**  Firstly, the asymptotical stability of (8) needs to be analyzed. Therefore, the purpose is to prove Φ<0 in (18).

Note that Φ can be rewritten as

(20) $$\begin{array}{c} \Phi =\mathrm{diag}{\left(\Omega_{i}\right)}_{i\in V}-\kappa T^{T}\left(\hat{L}⊗I_{n}\right)T \end{array}$$

where $\Omega_{i}=\left[\begin{array}{cc} \mathrm{Sym}(P_{io}{\bar{A}}_{io}-{\tilde{K}}_{io}{\bar{C}}_{io})+I & {\left(P_{iu}{\bar{A}}_{ir}\right)}^{T}\\ {}* & \mathrm{Sym}(P_{iu}{\bar{A}}_{iu})+I \end{array}\right]$.

Choose $r_{i}=r_{0}>0$ and $P_{iu}=I$ for i∈V. According to Lemma 2, there exists $0<\varepsilon <r_{0}$ satisfying (9). Hence,

(21) $$\begin{array}{c} \Phi <\mathrm{diag}{\left(\Omega_{i}\right)}_{i\in V}+\kappa \left(R-\varepsilon I\right) \end{array}$$

Select κ>0 large enough such that

(22) $$\begin{array}{c} {\bar{A}}_{iu}+{\bar{A}}_{iu}^{T}+I-\varepsilon \kappa +\frac{{\bar{A}}_{ir}{\bar{A}}_{ir}^{T}}{(r_{0}-\varepsilon )\kappa }<0 \end{array}$$

According to the Schur complement [28], (22) is equivalent to

(23) $$\begin{array}{c} \left[\begin{array}{cc} -(r_{0}-\varepsilon )\kappa & {\bar{A}}_{iu}^{T}\\ {}* & {\bar{A}}_{iu}+{\bar{A}}_{iu}^{T}+I-\varepsilon \kappa \end{array}\right]<0 \end{array}$$

Since the pair $\left({\bar{C}}_{io},{\bar{A}}_{io}\right)$ is observable, one can design $K_{io}=P_{io}^{-1}{\tilde{K}}_{io}$ such that ${\bar{A}}_{io}-K_{io}{\bar{C}}_{io}$ fulfils the Hurwitz criterion. Then, according to Lyapunov’s theorem, for an arbitrary matrix $Q_{i}<0$, there exists $P_{io}$ satisfying

(24) $$\begin{array}{c} \mathrm{Sym}\left(P_{io}\left({\bar{A}}_{io}-L_{io}{\bar{C}}_{io}\right)\right)+I+2\left(r_{0}-\varepsilon \right)\kappa I=0 \end{array}$$

Hence, ${\tilde{K}}_{io}=P_{io}K_{io}$. Substituting (24) into (23), it is obtained that $\Omega_{i}+\kappa \left(R_{i}-\varepsilon I\right)<0$ for i∈V, which, according to (21), yields Ω<0. Thus, the asymptotical stability of (8) is proven.

Divide an identity matrix $I_{N\bar{n}+\sum_{i=1}^{N}{\bar{q}}_{i}}$ into the form of

(25) $$\begin{array}{c} I_{N\bar{n}+\sum_{i=1}^{N}{\bar{q}}_{i}}=\mathrm{diag}\left\{I_{1}^{1},I_{1}^{2},\ldots ,I_{N}^{1},I_{N}^{2}\right\}, \end{array}$$

where $I_{i}^{j}$ has the same dimension as the *j*th diagonal block in $\Psi_{ii}$, for i=1,…,N and j=1,2.

Using the column description, $I_{N\bar{n}+\sum_{i=1}^{N}{\bar{q}}_{i}}$ can be rewritten as $I_{N\bar{n}+\sum_{i=1}^{N}{\bar{q}}_{i}}=\left[{\bar{I}}_{1}^{1},{\bar{I}}_{1}^{2},\ldots ,{\bar{I}}_{N}^{1},{\bar{I}}_{N}^{2}\right],$ where ${\bar{I}}_{i}^{j}$ has all zero entries except for $I_{i}^{j}$. By changing the position of the columns in $I_{N\bar{n}+\sum_{i=1}^{N}{\bar{q}}_{i}}$, an invertible matrix is defined as $I=[{\bar{I}}_{1}^{1},,\ldots ,{\bar{I}}_{N}^{1},{\bar{I}}_{1}^{2},\ldots ,{\bar{I}}_{N}^{2}]$. Then, pre-multiplying and post-multiplying $I^{T}$ and I on both sides of (11), respectively, one can obtain

(26) $$\begin{array}{c} \tilde{\Psi }=I^{T}\Psi I<0, \end{array}$$

where $\tilde{\Psi }=\left[\begin{array}{cc} \Phi & \Xi \\ {}* & -\gamma^{2}I \end{array}\right],$ with $\Xi =\left[\begin{array}{cc} P_{io}W_{io} & -{\tilde{K}}_{io}D_{i}\\ P_{iu}W_{iu} & 0 \end{array}\right]$.

Applying the Schur complement, it is concluded that Φ<0 if and only if one can select γ satisfying

$$\gamma >\sqrt{\lambda_{max}\left(-\Xi^{T}\Phi \Xi \right)}$$

Thus, the proof is completed. □

**Remark 1.**  *In this paper, beyond the $H_{\infty }$ suboptimal distributed observers presented in [8], an optimal solution for distributed observers with $H_{\infty }$ performance is proposed in this paper. The difference is because in [8], an $H_{\infty }$ performance index γ needs to be set in advance, while in this paper, γ can be maximized according to the optimization problem (14).*

**Remark 2.**  *Similar to the state x, the fault f in system (1) does not need to be fully estimated in each sensor node, while it can be cooperatively estimated by the observers equipped in the sensor networks. To accurately estimate x and f, the observability of the pair $\left(\mathrm{col}{\left({\bar{C}}_{i}\right)}_{i\in V},\bar{A}\right)$ other than the pair $\left({\bar{C}}_{i},\bar{A}\right)$ for i∈V is required, which is a much more relaxed condition.*

### 3.3. Fully Distributed Observers with Adaptive Gains

In this subsection, a special case of the process disturbance $d_{0}=0$ and the sensor disturbance $d_{i}=0$ for i∈V is considered, such that the fully distributed observers with adaptive gains are equipped. Subsequently, it is assumed that $\dot{f}=0$. The more general case that the *q*th-order derivate of the fault is zero can easily be extended to the case that $\dot{f}=0$ for q=2,3,…. This assumption makes sense from the perspective of engineering practices since many faults in applications are have a zero *q*th-order derivative [29].

Thus, the local estimation error of the observer in sensor node *i* is given as

(27) $${\dot{\bar{e}}}_{i}=\left(\bar{A}-K_{i}{\bar{C}}_{i}\right){\bar{e}}_{i}+H_{i}\sum_{j=1}^{N}a_{ij}\left({\bar{e}}_{j}-{\bar{e}}_{i}\right)$$

which corresponds to the following global error dynamics:

(28) $$\dot{\bar{e}}=\left(A-KC\right)\bar{e}-H\left(L⊗I_{\bar{n}}\right)\bar{e}$$

To implement the fully distributed design, matrices $K_{i}$ and $H_{i}$ in distributed observers (6) take the form of

(29) $$\begin{array}{c} K_{i}=T_{i}\left[\begin{array}{c} K_{io}\\ 0 \end{array}\right],\;H_{i}=\kappa_{i}T_{i}\left[\begin{array}{cc} 0 & 0\\ 0 & I \end{array}\right]T_{i}^{T}, \end{array}$$

where the gain $\kappa_{i}$ in each observer is designed as [30]

(30) $$\begin{array}{c} \kappa_{i}=\alpha_{i}+{\dot{\alpha }}_{i},\;{\dot{\alpha }}_{i}=\beta_{i}\psi_{i}^{T}\psi_{i},\;\psi_{i}=U_{i}^{T}\sum_{j\in N_{i}}l_{ij}{\hat{\bar{x}}}_{j},\;\alpha_{i}\left(0\right)>0,\;\beta_{i}>0 \end{array}$$

**Theorem 3.**  *The error dynamics (28) corresponding to the distributed observers (6) equipped with the adaptive law (30) is asymptotically stable if $K_{io}$ is designed such that ${\bar{A}}_{io}-K_{io}{\bar{C}}_{io}$ fulfils the Hurwitz criterion for ∀i∈V. Meanwhile, the adaptive gain $\kappa_{i}$ ultimately converges to some finite value.*

**Proof.**  Firstly, coordinate transformation is needed. Define

(31) $$\begin{array}{c} \left[\begin{array}{c} \xi_{i}\\ \psi_{i} \end{array}\right]=\left[\begin{array}{c} O_{i}^{T}{\bar{e}}_{i}\\ U_{i}^{T}\sum_{j=1}^{N}l_{ij}{\bar{e}}_{j} \end{array}\right] \end{array}$$

Then, the concatenated forms of $\xi_{i}$ and $\psi_{i}$ are $\xi =\mathrm{col}{\left(\xi_{i}\right)}_{i\in V}$ and $\psi =\mathrm{col}{\left(\psi_{i}\right)}_{i\in V}$, respectively, which, according to (27), correspond to the following coordinate transformation:

(32) $$\begin{array}{c} \left[\begin{array}{c} \xi \\ \psi \end{array}\right]=\left[\begin{array}{cc} I & 0\\ U^{T}\left(L⊗I_{n}\right)O & U^{T}\left(L⊗I_{n}\right)U \end{array}\right]{\left[\begin{array}{cc} O & U \end{array}\right]}^{T}\bar{e} \end{array}$$

Note that $U^{T}\left(L⊗I_{n}\right)U$ is nonsingular according to Lemma 2, and $\left[\begin{array}{cc} O & U \end{array}\right]$ is nonsingular as well. Then, it is deduced that the above coordinate transformation is nonsingular, which means that $\bar{e}$ is asymptotically stable if and only if ξ and ψ are asymptotically stable. In light of (28) and (32), the error dynamics of ξ and ψ is presented as

(33) $$\begin{array}{cc} \; & \dot{\xi }=A_{k}\xi \\ \; & \dot{\psi }={\bar{A}}_{r}\xi -U^{T}\left(L⊗I_{n}\right)U\left(\bar{\kappa }-A_{u}\right)\psi \end{array}$$

where $A_{k}=\mathrm{diag}{\left(A_{io}-K_{io}C_{io}\right)}_{i\in V}$, $\bar{\kappa }=\mathrm{diag}{\left(\kappa_{i}I_{\rho_{i}}\right)}_{i\in V}$, $A_{u}=\mathrm{diag}{\left(A_{iu}\right)}_{i\in V}$, ${\bar{A}}_{r}=U^{T}\left(L⊗I_{n}\right)OA_{k}+U^{T}\left(L⊗I_{n}\right)UA_{r}-U^{T}\left(L⊗I_{n}\right)UA_{u}{\left(U^{T}\left(L⊗I_{n}\right)U\right)}^{-1}U^{T}\left(L⊗I_{n}\right)O$, with $A_{r}=\mathrm{diag}{\left(A_{ir}\right)}_{i\in V}$.

Consider the dynamics (33). Since the pair $\left({\bar{C}}_{io},{\bar{A}}_{io}\right)$ is observable, one can design $K_{io}$ such that $A_{k}$ fulfils the Hurwitz criterion. Then, ξ is asymptotically stable, and the stability of ψ is determined by the following dynamics:

(34) $$\begin{array}{c} \dot{\psi }=-U^{T}\left(L⊗I_{n}\right)U\left(\bar{\kappa }-A_{u}\right)\psi \end{array}$$

Now, we dedicate ourselves to proving the asymptotical stability of dynamics (34). A Lyapunov function candidate is given as

(35) $$V_{1}=\frac{1}{2}\sum_{i=1}^{N}\theta_{i}\left(2\alpha_{i}+{\dot{\alpha }}_{i}\right){\dot{\alpha }}_{i}+\frac{1}{2}\sum_{i=1}^{N}\theta_{i}{\left(\alpha_{i}-\alpha_{i}^{*}\right)}^{2}$$

where $\alpha_{i}^{*}>0$ is to be determined.

Then, the time derivative of $V_{1}$ is obtained as

(36) $$\begin{array}{cc} {\dot{V}}_{1} & =\sum_{i=1}^{N}\theta_{i}{\dot{\alpha }}_{i}\beta_{i}\psi_{i}^{T}\psi_{i}+2\sum_{i=1}^{N}\theta_{i}\left(\alpha_{i}+{\dot{\alpha }}_{i}\right)\psi_{i}^{T}{\dot{\psi }}_{i}+\sum_{i=1}^{N}\theta_{i}\left(\alpha_{i}-\alpha^{*}\right)\beta_{i}\psi_{i}^{T}\psi_{i}\\ & =\psi^{T}\Phi \Theta B\psi -2\psi^{T}\left(M+\dot{M}\right)\Theta U^{T}\left(L⊗I_{n}\right)U\left(M+\dot{M}\right)\psi +2\psi^{T}\left(M+\dot{M}\right)\Theta U^{T}\left(L⊗I_{n}\right)UA_{u}\psi \\ & +\psi^{T}\left(M-g^{*}I\right)\Theta B\psi \\ = & \left[\begin{array}{cc} \psi^{T} & \left(M+\dot{M}\right)\psi^{T} \end{array}\right]\left[\begin{array}{cc} -g^{*}\Theta B & \Theta U^{T}\left(L⊗I_{n}\right)UA_{u}+\frac{\Theta B}{2}\\ {}* & -U^{T}\left(\hat{L}⊗I_{n}\right)U \end{array}\right]\left[\begin{array}{c} \psi \\ (M+\dot{M})\psi \end{array}\right] \end{array}$$

where $M=\mathrm{diag}{\left(\alpha_{i}I_{\rho_{i}}\right)}_{i\in V}$, $\dot{M}=\mathrm{diag}{\left({\dot{\alpha }}_{i}I_{\rho_{i}}\right)}_{i\in V}$, $\Theta =\mathrm{diag}{\left(\theta_{i}I_{\rho_{i}}\right)}_{i\in V}$, $B=\mathrm{diag}{\left(\beta_{i}I_{\rho_{i}}\right)}_{i\in V}$.

Thus, ${\dot{V}}_{1}<0$ if and only if

(37) $$\begin{array}{c} \left[\begin{array}{cc} -g^{*}\Theta B & \Theta U^{T}\left(L⊗I_{n}\right)UA_{u}+\frac{\Theta B}{2}\\ {}* & -U^{T}\left(\hat{L}⊗I_{n}\right)U \end{array}\right]<0 \end{array}$$

Note that $U^{T}\left(\hat{L}⊗I_{n}\right)U>0$ according to Lemma 2. According to the Schur complement, (37) is satisfied if $\alpha^{*}$ is selected satisfying

(38) $$\alpha^{*}>\frac{\lambda_{max}\left(\hat{\Omega }{\left(U^{T}\left(\hat{L}⊗I_{n}\right)U\right)}^{-1}{\hat{\Omega }}^{T}\right)}{\lambda_{min}\left(\Theta \right)\lambda_{min}\left(B\right)}$$

where $\hat{\Omega }=\Theta U^{T}\left(L⊗I_{n}\right)UA_{u}+\frac{\Theta B}{2}$.

Therefore, ${\dot{V}}_{1}<0$ is satisfied, meaning that *V* is bounded and thereby $\alpha_{i}$ is bounded. Meanwhile, considering that ${\dot{\alpha }}_{i}\geq 0$, it is induced that $\alpha_{i}$ converges to a finite value and ${\dot{\alpha }}_{i}=\beta_{i}\psi_{i}^{T}\psi_{i}$ converges to 0. Thus, it is concluded that $\kappa_{i}$ converges to a finite value and $\psi_{i}$ is asymptotically stable, which yields the asymptotical stability of $\bar{e}$ based on the above analysis. This ends the proof. □

**Remark 3.**  *From Theorem 3, one can see that the distributed observers (6) equipped with the matrices $K_{i}$ and $H_{i}$ in (29) can asymptotically estimate the state x and fault f. The parameter matrix $K_{io}$ is designed locally in each observer, and for the gain $\kappa_{i}$, only the relative estimation error associated with the neighboring node needs to be considered, as can be seen in (30). Thus, the observers (6) are designed in a fully distributed fashion.*

**Remark 4.**  *This section considers the special case of fully distributed observers for which no disturbances occur. Note that disturbances are usually unavoidable and an inevitable challenge in engineering applications. To handle this problem, the adaptive gains with the σ modification technique [31] can be used to realize robust fully distributed observers in the presence of two-norm bounded disturbances.*

## 4. Case Studies: A Spacecraft Attitude Control System

### 4.1. Example 1: Robust Distributed Observers with $H_{\infty }$ Performance

**Figure 2:**
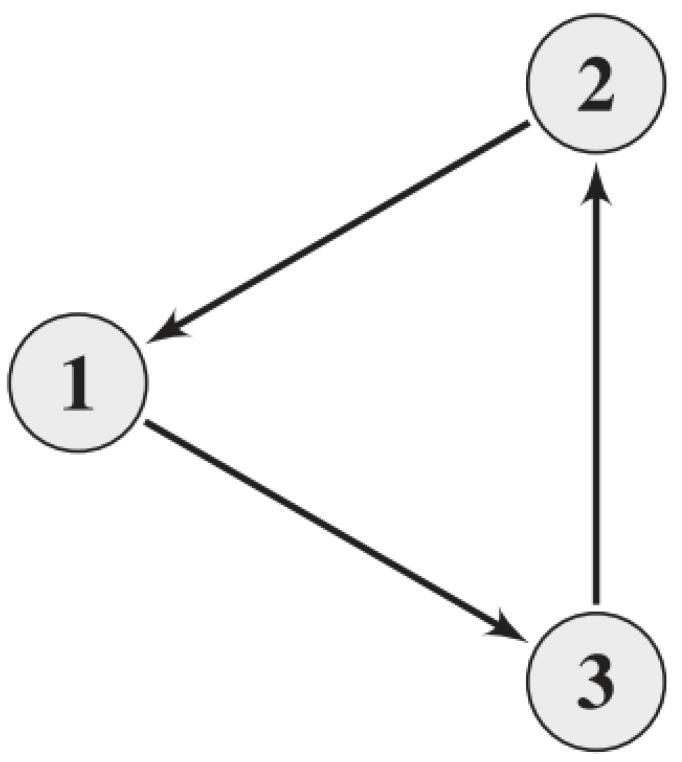
Communication topology of the sensor networks.

For the first example, simultaneous state and fault estimation for the spacecraft attitude control system is considered to verify the proposed robust distributed observers with $H_{\infty }$ performance. Consider a rigid spacecraft with its attitudes monitored by a network of 3 sensors, for which the communication topology is shown in Figure 2. Thus, the corresponding Laplace matrix is $L=\left[\begin{array}{ccc} 1 & -1 & 0\\ 0 & 1 & -1\\ -1 & 0 & 1 \end{array}\right]$ with $\theta_{1}=\theta_{2}=\theta_{3}=\frac{1}{3}$. Denote $x={\left[\begin{array}{cccccc} x^{1} & x^{2} & x^{3} & x^{4} & x^{5} & x^{6} \end{array}\right]}^{T}$ as the state of the spacecraft attitude control system, with components $x^{1}$, $x^{2}$ and $x^{3}$ representing the attitude angles of each axis and components $x^{4}$, $x^{5}$ and $x^{6}$ denoting the corresponding attitude angular velocities, respectively. Under the small range of attitude changes, the system can be linearized as a second-order system in the form of (1), which, according to [32], has the following parameter matrices:

$A=\left[\begin{array}{cccccc} 0 & 0 & 0 & 1 & 0 & 0\\ 0 & 0 & 0 & 0 & 1 & 0\\ 0 & 0 & 0 & 0 & 0 & 1\\ \frac{J_{y}-J_{z}}{J_{x}}\omega_{0}^{2} & 0 & 0 & 0 & 0 & \frac{J_{y}-J_{z}-J_{x}}{J_{x}}\omega_{0}\\ 0 & 0 & 0 & 0 & 0 & 0\\ 0 & 0 & \frac{J_{y}-J_{z}}{J_{z}}\omega_{0}^{2} & \frac{J_{y}-J_{z}-J_{x}}{J_{z}}\omega_{0} & 0 & 0 \end{array}\right]$, $B=\left[\begin{array}{ccc} 0 & 0 & 0\\ 0 & 0 & 0\\ 0 & 0 & 0\\ \frac{1}{J_{x}} & 0 & 0\\ 0 & \frac{1}{J_{y}} & 0\\ 0 & 0 & \frac{1}{J_{z}} \end{array}\right]$, $C_{1}=\left[\begin{array}{cccccc} 1 & 0 & 0 & 0 & 0 & 0\\ 0 & 0 & 0 & 1 & 0 & 0 \end{array}\right]$, $C_{2}=\left[\begin{array}{cccccc} 0 & 1 & 0 & 0 & 0 & 0\\ 0 & 0 & 0 & 0 & 1 & 0 \end{array}\right]$, $C_{3}=\left[\begin{array}{cccccc} 0 & 0 & 1 & 0 & 0 & 0\\ 0 & 0 & 0 & 0 & 0 & 1 \end{array}\right]$. where $J_{x}=0.0109$, $J_{y}=0.0506$ and $J_{z}=0.0509$ together form the moment of inertia $J=\mathrm{diag}(J_{x},J_{y},J_{z})$, and $\omega_{0}=-2\pi$ is the orbital angular velocity. The focus of the paper is not on the design of the control law itself but rather on the distributed state and fault estimation approach we present. So, the control input can be arbitrarily designed and is here selected as a state-feedback (or an observer-based output-feedback) controller such that the states satisfy marginal stability in the absence of disturbances or faults. Then, the remaining parameter matrices in (1) are given as

$$\begin{array}{c} \begin{array}{cc} \; & E_{0}=\left[\begin{array}{ccc} 0 & 0 & 0\\ 0 & 0 & 0\\ 0 & 0 & 0\\ 1 & 0 & -1\\ 2 & 1 & 0\\ 0 & 0.5 & 1 \end{array}\right],D_{0}=\left[\begin{array}{ccc} 1 & 0 & -10\\ 0 & 1 & 1\\ 2 & 0.5 & 1.5\\ 0 & 0 & 0\\ 0 & 0 & 0\\ 0 & 0 & 0 \end{array}\right]\\ \; & E_{1}=\left[\begin{array}{c} 000\\ 100 \end{array}\right],E_{2}=\left[\begin{array}{c} 000\\ 010 \end{array}\right],E_{3}=\left[\begin{array}{c} 000\\ 001 \end{array}\right],D_{1}=D_{2}=D_{3}=\left[\begin{array}{c} 1\\ 0 \end{array}\right]. \end{array} \end{array}$$

By solving the optimization problem (14), the $H_{\infty }$ performance index is minimized as γ=1.7324, with the scalar in (6) given as $\kappa =3.186\times 10^{5}$. The initial value of the states is selected as $x\left(0\right)={\left[\begin{array}{cccccc} 3 & 4 & 2 & -5 & 2 & 0 \end{array}\right]}^{T}$. In the simulation, the process disturbances are selected as $d_{0}={\left[\begin{array}{ccc} \sin(t) & \sin(t) & \sin(t) \end{array}\right]}^{T}$, and the measurement disturbances are $d_{1}=d_{2}=d_{3}=0.5\cos\left(t\right)$.

The injected time-varying faults $f={\left[\begin{array}{ccc} f^{1} & f^{2} & f^{2} \end{array}\right]}^{T}$ are in the form of

$f^{1}=\left\{\begin{array}{cc} 0 & t<2\\ 10\sin\left(1.5t-3\right)+5\cos\left(3t-6\right) & t\geq 2 \end{array}\right.$,

$f_{2}=\left\{\begin{array}{cc} 0 & t<4\\ 4t-16 & 4\leq t<8\\ 16 & 8\leq t<12\\ 20e^{24-2t}-4 & t\geq 12 \end{array}\right.$,

$f_{3}=\left\{\begin{array}{cc} 0 & t<4\\ 10e^{2-0.5t}-10 & t\geq 4 \end{array}\right.$.

**Figure 3:**
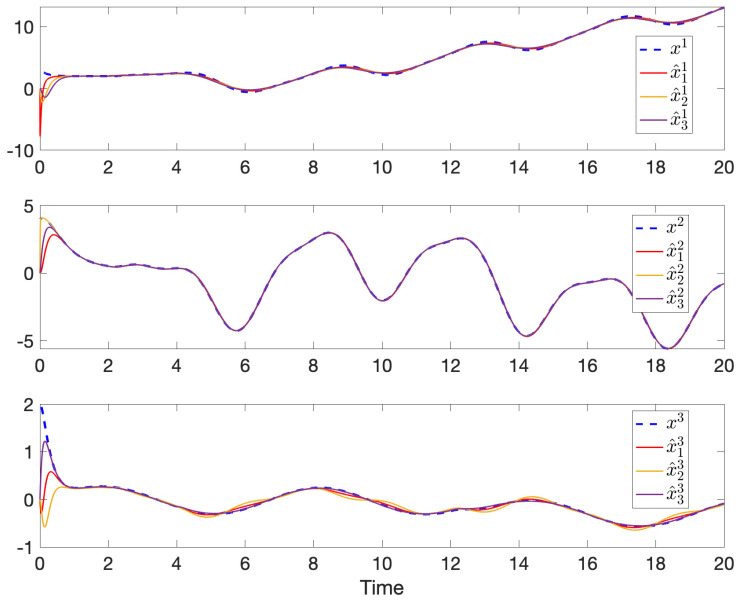
The attitude angles $x^{1}$, $x^{2}$, and $x^{3}$ and their estimations in each sensor.

**Figure 4:**
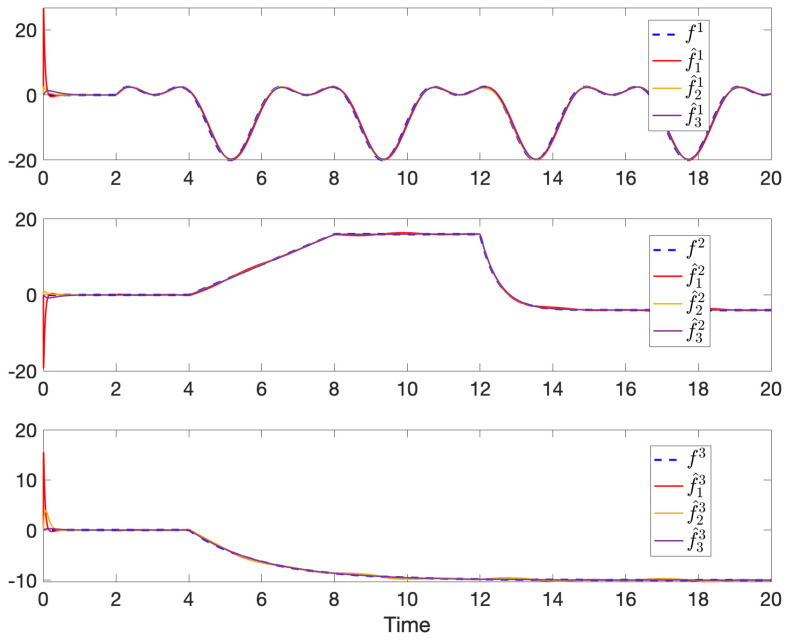
The time-varying faults $f^{1}$, $f^{2}$, and $f^{3}$ and their estimations in each sensor.

The simulation results of robust state and fault estimation for the spacecraft attitude control system are shown in Figure 3 and Figure 4. One can see that the attitude angles and the injected time-varying faults can be successfully estimated by all observers with the existence of both process disturbances and measurement disturbances. Thus, the properties of asymptotic estimation and disturbance attenuation are illustrated.

### 4.2. Example 2: Fully Distributed Observers with Adaptive Gains

The second example is extended to verify the proposed fully distributed observers for the spacecraft attitude control system. The control input is selected as a state-feedback (or an observer-based output-feedback) controller such that the states satisfy asymptotical stability. Consider the same values for the system’s parameter matrices as in Example 1. Here, the initial value of the states is selected as $x\left(0\right)={\left[\begin{array}{cccccc} 1 & 2 & -3 & 0.5 & -0.3 & 0.2 \end{array}\right]}^{T}$. The parameters in the adaptive gains are $\beta_{1}=\beta_{2}=\beta_{3}=1$, and the initial values of $\alpha_{i}$ for i∈V are selected as $\alpha_{1}\left(0\right)=\alpha_{2}\left(0\right)=\alpha_{3}\left(0\right)=0.1$. The faults are selected as constant values, that is, $f={\left[\begin{array}{ccc} 2 & -3 & 4 \end{array}\right]}^{T}$, to represent the stuck faults frequently occurring in the spacecraft attitude control system. Disturbances are assumed not to exist.

**Figure 5:**
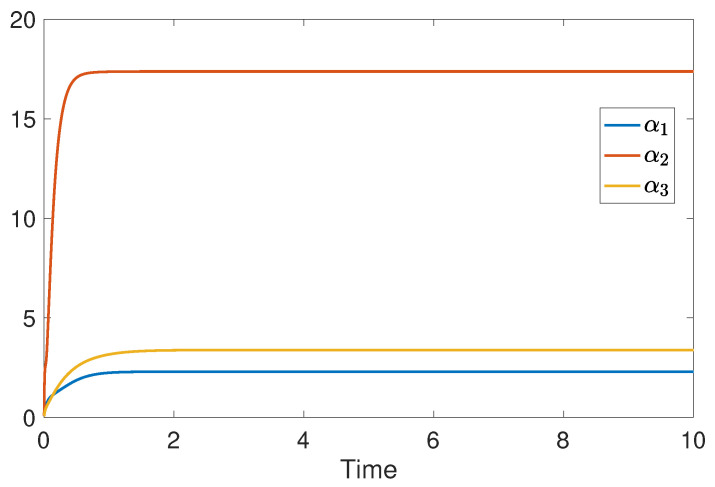
The values of $\alpha_{1}$, $\alpha_{2}$ and $\alpha_{3}$.

**Figure 6:**
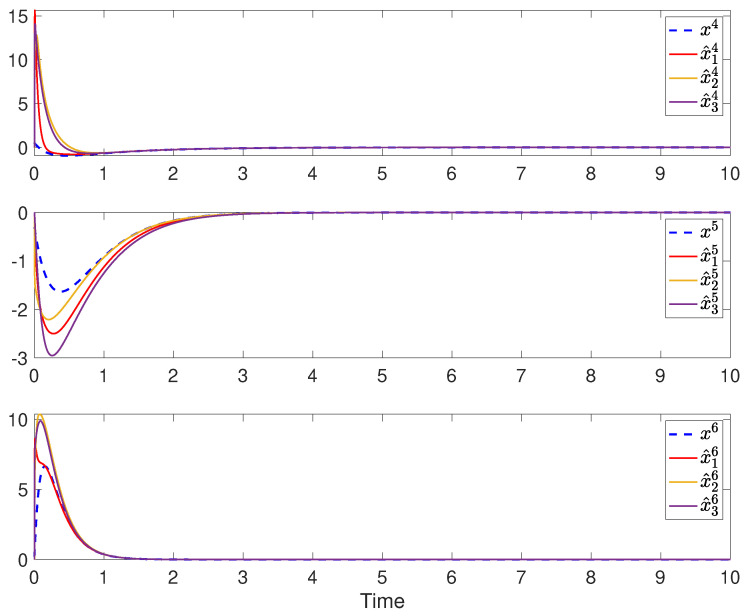
The attitude angular velocities $x^{1}$, $x^{2}$ and $x^{3}$ and their estimations in each sensor.

**Figure 7:**
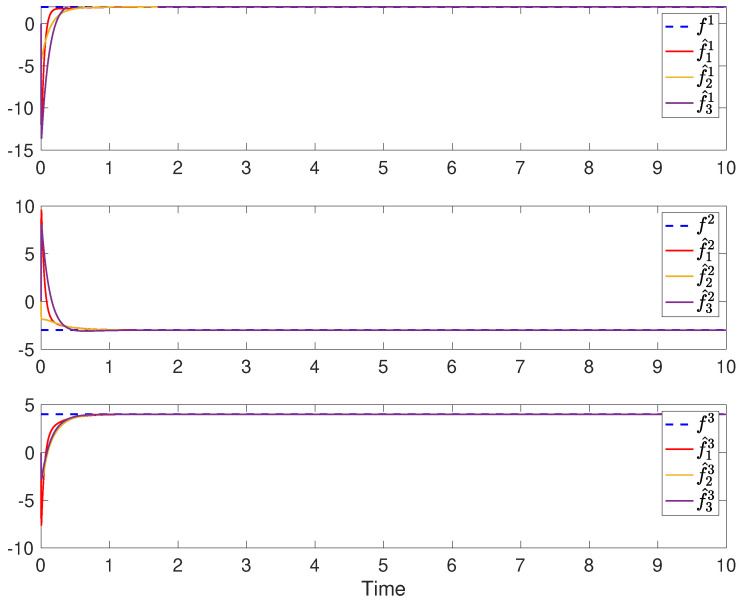
The time-varying faults $f^{1}$, $f^{2}$ and $f^{3}$ and their estimations in each sensor.

The simulation results of fully distributed state and fault estimation for the spacecraft attitude control system are shown in Figure 5, Figure 6 and Figure 7. In Figure 5, it can be found that $\alpha_{i}$ for i∈V converges to some finite value ultimately. Then, one can see from Figure 6 and Figure 7 that the attitude angular velocities and the injected constant faults can be asymptotically estimated by all observers in the absence of disturbances. Moreover, in light of (30), it can be deduced that ${\dot{\alpha }}_{i}$ asymptotically converges to 0 according to Figure 6 and Figure 7. Therefore, it is concluded that the adaptive gain $\kappa_{i}$ ultimately converges as well. Thus, the properties of the proposed fully distributed observers are illustrated.

## 5. Conclusions

In this paper, distributed observers monitoring an LTI system have been proposed, which can estimate the system states and faults based on sensor networks using only local measurements and local interactions with neighbors. The distributed observers do not require local observability of any sensor node in terms of the system state and faults but rather collective observability of all sensors. $H_{\infty }$ performance has been introduced for the distributed observers, which has guaranteed the estimation error is robust to disturbances. The global design condition of the $H_{\infty }$ performance was transformed into a solvable LMI given the collective observability of the system and a suitable $H_{\infty }$ performance index. To make the observer designed fully distributed, adaptive gains have been considered, which means the states and faults can be asymptotically estimated by each observer in the absence of disturbances. Case studies on a spacecraft attitude control system were studied to illustrate the effectiveness and capabilities of the proposed method. Specifically, both the state and the faults can be estimated relatively accurately by the robust distributed observer designed in each node in the presence of disturbances. Meanwhile, in the case that no disturbances exist, the fully distributed observers can realize asymptotical estimation, with the adaptive gains ultimately converging to certain finite values. The disadvantage of the proposed method is that disturbances are not considered for the fully distributed observers, which usually occur in engineering applications. For the $L_{2}$-norm bounded disturbance, attenuation using fully distributed observers with $H_{\infty }$ performance is considered a future research direction. Future work may also focus on the performance of the distributed observers under cyber attacks, especially the different influences of physical faults and cyber attacks on the distributed observers.

## Data Availability

The original contributions presented in the study are included in the article.
